# The impact of extracorporeal diaphragm pacing therapy on diaphragm function in critically III patients dependent on mechanical ventilation: a double-blind randomized controlled trial

**DOI:** 10.1097/MD.0000000000045157

**Published:** 2025-11-07

**Authors:** Linru Qiao, Zhen Yang, Shangang Zhang, Zhixia Zhang, Mengli Xu

**Affiliations:** a Department of Rehabilitation, Tianyou Hospital, Wuhan University of Science and Technology, Wuhan, China; b Department of Nursing, Tianyou Hospital, Wuhan University of Science and Technology, Wuhan, China.

**Keywords:** diaphragm function, Extracorporeal Diaphragm Pacing, ventilator dependent

## Abstract

**Background:**

To evaluate the impact of extracorporeal diaphragm pacing (EDP) therapy on diaphragm function in critically ill patients reliant on mechanical ventilation.

**Methods:**

Patients were randomly divided into an observation group (39 cases) and a control group (40 cases) using a random number table. Both groups received bedside rehabilitation interventions. Additionally, the observation group underwent EDP therapy 6 times weekly for 2 weeks. Diaphragm activity, diaphragm thickness, arterial oxygen tension (PaO_2_), arterial carbon dioxide tension (PaCO_2_), and Oxygenation Index (OI) were measured at baseline and 2 weeks postenrollment or upon extubation.

**Results:**

Both groups showed significant improvements in diaphragm activity, PaO_2_, PaCO_2_, and OI compared to baseline (*P* <.05). The observation group had significantly better outcomes in diaphragm thickness (0.22 ± 0.03), diaphragm activity (13.96 ± 0.73), PaCO2 (37.59 ± 5.66), and OI (271.10 ± 17.25) compared to the control group (0.20 ± 0.02, 12.24 ± 0.99, 47.25 ± 5.64, 248.60 ± 15.26), with statistical significance (*P* <.05).

**Conclusion:**

EDP therapy offers benefits in improving diaphragm function in critically ill patients on mechanical ventilation. It enhances diaphragm strength and endurance, increases diaphragm activity, improves pulmonary ventilation, and reduces mechanical ventilation duration.

**Key practice points**

1. Extracorporeal diaphragm pacing (EDP) therapy can maintain respiratory function in patient’s dependent on mechanical ventilation.
2. EDP therapy is recommended to improve lung function in elderly patients who are bedridden for extended periods.
3. For critically ill patients in the intensive care unit (ICU) on mechanical ventilation, EDP therapy is advised to prevent potential ventilator dependency.

## 1. Introduction

Research indicates that the incidence of ventilator-induced diaphragmatic dysfunction (VIDD) among mechanically ventilated patients ranges from 40% to 60%.^[1]^ Among patients undergoing readiness assessments for extubation, approximately 29% exhibit diaphragmatic dysfunction.^[2]^ This phenomenon primarily stems from the excessive support provided by mechanical ventilation or profound sedation levels, both of which contribute to reduced respiratory muscle strength and subsequent muscle atrophy.^[3,4]^ Furthermore, the diaphragm itself is highly susceptible to disuse atrophy, with studies showing that the occurrence of diaphragmatic weakness in critically ill patients is twice that of limb muscle weakness.^[5]^ VIDD significantly compromises the success rate of weaning from mechanical ventilation, prolongs ventilation duration, and leads to respiratory dependency.

Diaphragmatic weakness refers to the inability of the diaphragm to achieve its maximum normal strength, with a notably high incidence among critically ill patients. Possible mechanisms include: mechanical ventilation, by completely or partially taking over spontaneous breathing, prevents autonomous diaphragmatic contraction, leading to decreased muscle strength and atrophy,^[6]^ Both excessive and inadequate ventilatory support can cause diaphragmatic injury, thereby impairing diaphragm function; studies indicate that compared to other skeletal muscles, the diaphragm shows higher expression of growth-inhibiting genes under unloaded conditions.^[7]^ This results in more pronounced reductions in muscle fiber density, lower efficiency in synthesizing myosin and actin heavy chains, and impaired excitation-contraction coupling function. Following disuse atrophy of the diaphragm, heightened activity of muscle proteinase systems increases fiber protein breakdown, accelerates apoptosis of muscle fiber cells, and exacerbates damage under hypoxic conditions.^[8]^ The duration of mechanical ventilation correlates positively with the extent of diaphragmatic impairment; as ventilation time lengthens, symptoms of diaphragmatic weakness progressively worsen. Animal studies have observed abnormal ultrastructural changes in rat diaphragms at different durations of mechanical ventilation, with increasing severity and proportion of abnormalities over time.^[9]^

EDP is a therapeutic approach placed externally on the body that utilizes low-frequency pulse mode electrical stimulation to exercise respiratory muscles. It induces contractions of diaphragm motor units, positively regulating respiratory system function by stimulating the phrenic nerve. This method improves pulmonary ventilation while maintaining diaphragmatic strength and endurance.^[10]^ Currently, EDP is gradually becoming more prevalent in clinical practice due to its noninvasive, safe, and user-friendly characteristics,^[11]^ though its efficacy still requires further validation through additional clinical trials. This study applied EDP therapy to critically ill mechanically ventilated patients and observed significant improvements in diaphragm function, demonstrating satisfactory clinical efficacy.

## 2. Methods

### 2.1. Participants

Between March 2021 and March 2024, 86 patients meeting the aforementioned criteria were enrolled from the Intensive Care, Neurosurgery, and Respiratory and Critical Care Medicine departments. Patients were randomly assigned into 2 groups: an observation group and a control group, each consisting of 43 individuals. Inclusion criteria mandated primary critical illness in the acute phase with a glasgow coma scale (GCS) score ≤8, meeting mechanical ventilation criteria,^[12]^ and having received at least 72 hours of ventilatory assistance. Additionally, patients exhibited diaphragmatic dysfunction,^[13]^ characterized by diaphragm thickness <2 mm, diaphragm thickening fraction <20%, and diaphragm excursion <10 mm. Skin near the sternocleidomastoid muscle was intact and adequately exposed, with an anticipated hospital stay of ≥ 2 weeks. Patients were aged between 18 and 80 years, and informed consent was obtained from patients or their legal representatives. Exclusion criteria included a history of chronic respiratory diseases, severe respiratory diseases or infectious pleural diseases, newly diagnosed rib fractures, cardiac pacemakers or other implants, damaged diaphragmatic structure precluding ultrasound assessment, and unstable vital signs despite advanced life support measures. Baseline characteristics such as gender, age, BMI index, GCS score, and Acute Physiology and Chronic Health Evaluation (APACHE II) scores were statistically comparable (*P* >.05). All patients or their representatives provided informed consent for the clinical study, which was approved by the hospital’s Medical Ethics Committee (Approval No.: LL2024-04-22-01). This study has been retrospectively registered with the Chinese Clinical Trial Registry (ChiCTR2400092389).

### 2.2. Intervening measures

All patients underwent continuous monitoring of vital signs, blood oxygen saturation, and fluid balance. They received treatment plans developed by specialist physicians tailored to their primary conditions, including medications for the respiratory system such as antibiotics, nebulization, mucolytics, and bronchodilators, among others. Comprehensive nursing care was provided according to advanced care standards, medical orders, and patient needs, encompassing specialized care such as oral care, turning and positioning, negative pressure suctioning for sputum, tracheostomy/intubation care, and ventilator maintenance.^[14]^

Additionally, all patients underwent bedside rehabilitation interventions, including passive strengthening exercises for limb muscles, passive range of motion exercises for limb joints, pulmonary rehabilitation training, and physical therapy involving modalities including medium and low-frequency treatments. Given that enrolled patients were comatose, targeted pulmonary rehabilitation focused on airway clearance principles, employing positional drainage, mechanical sputum clearance, assisted breathing techniques, and cough facilitation methods. These rehabilitation interventions were administered once daily, 6 times per week, continuously for 2 weeks.^[15]^

In the observation group, patients received EDP treatment using the Guangzhou-produced EDP device (model HLO-GJ13A). Prior to treatment, patients were positioned supine with the head of the bed elevated by 30°, and the skin at the electrode sites was cleaned with water or alcohol. Subsequently, electrodes were connected, the device was activated, and the intensity was initially set to 0. Two small pacing electrodes were placed at the lower third of the outer edge of the sternocleidomastoid muscle, approximately at the shallowest part of the phrenic nerve. Additionally, 2 large auxiliary electrodes were positioned at the intersection between the midline of the clavicle and the second intercostal space. During treatment, electrical stimulation commenced during the inhalation phase, with a pacing rate of 12 times per minute and a pulse frequency of 40 ± 2.5 Hz. The stimulation intensity was gradually increased, typically ranging from 8 to 14 units, adjusted based on patient tolerance. Each treatment session lasted for 30 minutes, administered once daily, 6 times per week, continuously for 2 weeks.

### 2.3. Assessment method

#### 2.3.1. Diaphragm motion and diaphragm thickness measurement

Using the Dutch-manufactured portable ultrasound machine (Philips Purewave M model), prior to testing, patients were maintained in CPAP/PSV ventilator mode with an end-expiratory positive airway pressure of 4 cmH_2_O and a tidal volume of 6 to 8 mL/kg to ensure stable breathing without respiratory distress. Patients were positioned supine with the head of the bed elevated approximately 15° to 30°. The convex array probe was placed along the midline of the right clavicle or the anterior axillary line at the 7th-9th intercostal spaces. In 2D mode, using the liver as an acoustic window, the probe was directed towards the head and back, displaying 2 parallel waveform images with the diaphragm structure visible between them. Diaphragm thickness was measured as the distance between these 2 layers.^[16]^ For measuring diaphragm excursion using the M-mode probe, the sample line of the M-mode probe was directed towards the top of the diaphragm, with an angle of <30° to the long axis, positioned as vertically as possible across the 2 parallel waveform layers. Diaphragm excursion was measured during both expiration and inspiration, with the difference between the 2 representing diaphragm excursion.^[17]^ Results were observed and recorded after 3 respiratory cycles, selecting the maximum value obtained.

#### 2.3.2. Arterial blood gas analysis evaluation

All patients underwent arterial blood sampling from the peripheral artery both prior to enrollment and 2 weeks after enrollment. Blood gas analysis was performed using a Spanish-manufactured blood gas analyzer (Woffin GEM3500) to measure arterial oxygen partial pressure (PaO_2_), carbon dioxide partial pressure (PaO_2_), and oxygenation index (OI). Additionally, mechanical ventilation duration and intensive care unit (ICU) stay were recorded for each patient.

Furthermore, assessments were conducted at both preenrollment and 2 weeks postenrollment. For patients successfully weaned from mechanical ventilation and restored to spontaneous breathing, assessments were performed on the day of extubation.

The assessment results were conducted under single-blind conditions, where all assessors received standardized training and demonstrated competency through evaluation.

### 2.4. Sample size

The sample size was assessed based on non-inferior clinical trials and established by considering the Diaphragm motion and diaphragm thickness measurement as a primary outcome and was based on the following assumptions: significance level (α) = 0.05,type 2 error (β) = 0.2, and 90% test power. For sample calculation, G * Power 3.1.9 was used based on an effect size of 0.9. The calculated sample size was 76 patients per group. Considering a 5% dropout rate, a minimum total sample of 84 patients (42 per group) was required.

### 2.5. Statistical methods

All data were statistically analyzed using SPSS version 29.1. Normality and homogeneity of variance were assessed using the Kolmogorov-Smirnov (K-S) test and Levene test, respectively. For normally distributed data, paired t-tests were used for within-group comparisons before and after treatment, and independent sample t-tests were used for between-group comparisons. Non-normally distributed data were analyzed using the Mann–Whitney U test for between-group comparisons and the Wilcoxon Signed-Rank test for within-group comparisons before and after treatment. Categorical data comparisons were conducted using the x^2^ chi-square test. Statistical significance was set at *P* <.05 for all tests, using 2-tailed analysis.

## 3. Results

**Figure 1.**
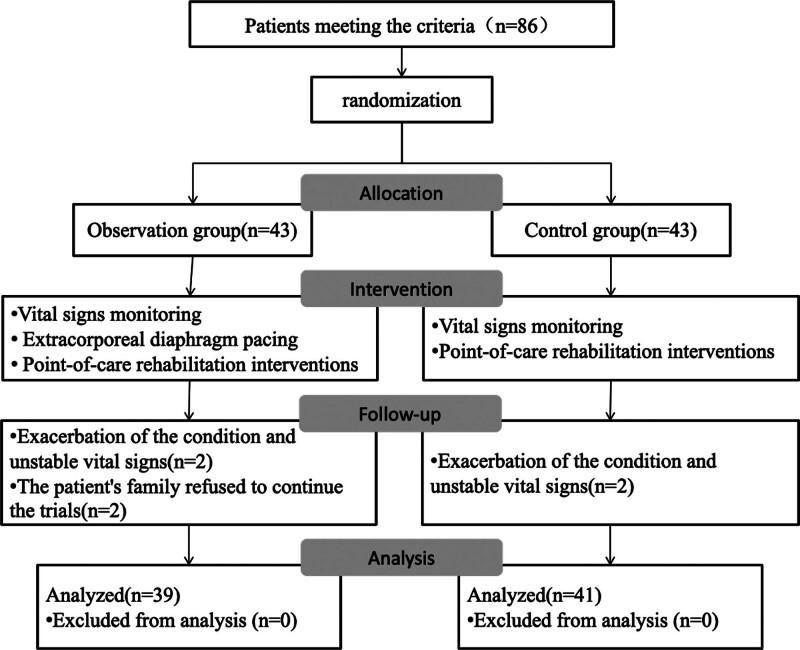
Research flowcharts. A total of 86 patients who met the inclusion criteria were randomly assigned to either the observation group (n = 43) or the control group (n = 43). The observation group received vital signs monitoring, EDP, and point-of-care rehabilitation interventions. The control group received vital signs monitoring and point-of-care rehabilitation interventions only. During follow-up, 2 patients in each group were excluded due to exacerbation of their condition with unstable vital signs. Additionally, 2 patients in the observation group withdrew from the study upon their families’ requests. Ultimately, 39 patients in the observation group and 41 in the control group completed the study and were included in the final analysis, with no patients excluded during the analysis phase. EDP = extracorporeal diaphragm pacing.

Figure 1 illustrates the study flowchart based on the Consolidated Standards of Reporting Trials (CONSORT) guidelines. A total of 86 patients were randomly allocated, with 80 patients completing the experiment. Six patients dropped out: 2 from the observation group due to disease exacerbation, and 2 from each group due to patient family refusal, leaving 39 patients completing the treatment in the observation group and 41 in the control group.

Table 1 presents demographic data including gender, age, BMI, GCS score, and APACHE II score of enrolled patients. Statistical analysis showed no significant differences between the 2 groups before enrollment (*P* >.05), indicating comparability.

**Table 1:** General information of enrolled patients.

Measures	Observation group (n = 39)	Control group (n = 41) M(SD)	t*/P*
	M(SD)		
Age	64.82 (9.01)	60.43 (7.86)	−0.79/.43
BMI index	6.95 (2.54)	6.45 (2.61)	2.31/.24
GCS scoring	4.79 (1.05)	4.90 (1.08)	−0.68/.54
APACHE Ⅱ scoring	17.56 (2.95)	19.58 (2.96)	−3.02/.11
	Percent(n)	Percent(n)	X^2^*/P*
Gender			
Male	59 (23)	50 (20)	0.641/.50
Female	41 (16)	50 (20)	

APACHE II = acute physiology and chronic health evaluation, BMI = body mass index, GCS = glasgow coma scale, SD = standard deviation.

Table 2 compares diaphragm thickness and diaphragm excursion between the 2 groups. Before treatment, there were no significant differences in diaphragm thickness or excursion between the 2 groups (*P* >.05). Posttreatment, both diaphragm excursion in the observation group and diaphragm thickness in the control group showed significant improvements compared to pretreatment (*P* <.05). Between-group comparisons after treatment revealed significantly higher diaphragm thickness and excursion in the observation group compared to the control group (*P* <.05).

**Table 2:** Comparison of diaphragm activity, diaphragm thickness, and blood gas results between the 2 groups.

Outcome	Observation group (n = 39)	Control group (n = 41)	*t*	*P*
	M(SD)	M(SD)		
Diaphragm thickness (cm)				
Pretreatment	0.20 (0.03)	0.19 (0.02)	0.61	.54
Posttreatment	0.22 (0.03)*,**	0.20 (0.02)	4.23	**<**.001
Diaphragmatic motion(cm)				
Pretreatment	11.18 (0.93)	11.06 (1.31)	0.49	.63
Posttreatment	13.96 (0.73)*,**	12.24 (0.99)*	8.71	**<**.001
Pa0_2_(mm Hg)				
Pretreatment	67.77 (7.37)	67.18 (3.62)	0.45	.65
Posttreatment	71.97 (2.71)*	71.83 (5.38)*	0.15	.87
PaC0_2_(mm Hg)				
Pretreatment	50.85 (9.02)	56.30 (7.21)	−2.93	.09
Posttreatment	37.59 (5.66)*,**	47.25 (5.64)*	−7.22	**<**.001
IO (mm Hg)				
Pretreatment	204.59 (28.32)	202.45 (24.11)	0.36	.71
Posttreatment	271.10 (17.25)*,**	248.60 (15.26)*	6.15	**<**.001

Compared between the observation group and control group, compared with pretreatment within the group.

* *P* <.05; compared with the control group posttreatment.

** *P* <.05.

Table 2 also compares arterial blood gas analysis results between the 2 groups of patients. Before treatment, there were no significant differences in Pa0_2_, PaC0_2_, and OI between the 2 groups (*P* >.05). Posttreatment, both groups showed significant improvements in Pa0_2_, PaC0_2_, and OI compared to pretreatment (*P* <.05). Between-group comparisons after treatment indicated that the observation group had significantly lower Pa0_2_ and higher OI compared to the control group (*P* <.05). For detailed values, refer to Table 2.

Patients in the observation group experienced significantly shorter durations of mechanical ventilation and ICU stay compared to the control group, with statistical significance (*P* <.05). For detailed values, refer to Table 3.

**Table 3:** Comparison of mechanical ventilation time and stay days between 2 groups.

Groups	Cases	Mechanical ventilation time (days)	Length of stay (days)	t*/P*
		M(SD)	M(SD)	
Observation group	39	10.00 (3.61)*	13.74 (2.57)*	−2.16/.03
Control group	41	12.15 (5.10)	16.73 (3.81)	−5.42/**<**.001

Compared with the control group after treatment.

* *P* <.05.

## 4. Discussion

Research indicates that the primary cause of diaphragm weakness in patients undergoing mechanical ventilation is the duration of mechanical ventilation itself.^[18]^ Therefore, shortening mechanical ventilation time through various interventions has become a focal point in current research. Diaphragm pacing involves rhythmic contraction of the diaphragm through electrical stimulation of the phrenic nerve, resembling physiological respiratory movements. EDP, known for its simplicity and feasibility, has been proven safe and effective both domestically and internationally. However, previous studies have primarily focused on chronic conditions such as cervical spinal cord injury, chronic obstructive pulmonary disease, pulmonary heart disease, and refractory hiccups.^[19]^ In conjunction with this study, EDP demonstrates significant advantages in the treatment of critically ill patients dependent on mechanical ventilation. These advantages are primarily manifested in the following aspects:

### 4.1. Ensuring diaphragm thickness and contractile ability

Patients in critical care units often experience consciousness disorders due to various critical illnesses, and mechanically ventilated patients often require sedation to reduce ventilator dyssynchrony. During this time, although the phrenic nerve remains intact, the electromyographic signals fail to induce diaphragm contraction, ultimately leading to disuse atrophy of the diaphragm. Previous studies have shown that both groups of patients exhibited lower diaphragm thickness and reduced diaphragm activity before treatment, indicating varying degrees of diaphragm dysfunction. EDP can recruit atrophied motor units, enhance muscle fiber capability, and maintain a relatively normal proportion of muscle fibers, thereby ensuring diaphragm function.^[20]^ Following intervention in the study, the observation group showed significantly improved diaphragm thickness and activity compared to the control group, consistent with earlier research findings.^[13,21]^

### 4.2. Improving ventilation capacity

The mobility of the diaphragm is closely linked to lung ventilation function, and prolonged mechanical ventilation leads to rapid declines in the strength and endurance of respiratory muscle groups, including the diaphragm, thereby reducing gas exchange efficiency and impacting lung ventilation function.^[22]^ Before treatment, both groups of patients in the study exhibited varying degrees of hypoxia and CO_2_ retention. EDP enhances diaphragm activity through stimulation, ensuring respiratory gas exchange with external pressures, enhancing lung compliance, and improving lung ventilation function. Following intervention, the observation group demonstrated superior blood gas results compared to the control group.

### 4.3. Shortening ventilation time and ICU stay

VIDD not only complicates weaning from mechanical ventilation, prolonging ventilation time, but also increases the risk of lung infections and other systemic complications, thereby extending ICU stay and potentially leading to respiratory failure and increased mortality risk.^[23]^ Therefore, early extubation after stabilizing the patient’s condition holds significant prognostic benefits. In this study, the observation group exhibited an average reduction in mechanical ventilation time by approximately 2 days and a reduction in ICU stay by approximately 3 days compared to the control group. Additionally, during the study, the observation group consistently outperformed the control group in weaning trials. However, clinical outcomes in critically ill patients are influenced by primary diseases, underlying conditions, physical status, and family expectations, which may introduce biases, thus limiting the generalizability of the results.

Innovatively, this trial demonstrates that EDP therapy significantly enhances diaphragm function and respiratory parameters in critically ill patients, which can lead to earlier weaning from mechanical ventilation. This improvement in diaphragm strength and endurance may positively impact the overall prognosis and recovery trajectory of these patients, potentially reducing ICU length of stay and associated healthcare costs. Furthermore, by restoring diaphragm function, EDP therapy can minimize the risks of VIDD, which is a critical concern in prolonged mechanical ventilation. As a result, integrating EDP therapy into standard care protocols could serve as a promising strategy to optimize respiratory rehabilitation and improve patient outcomes in clinical practice.

Nevertheless, this study has several limitations: (i) the heterogeneity of enrolled patients and variability in disease severity may introduce outcome biases; (ii) assessments were conducted only at baseline, postintervention, or after weaning, without continuous monitoring to observe trends in outcome measures; (iii) stimulation intensity and methods were based on empirical evidence and previous studies, without different intervention groups for comparison; (iv) patients were admitted to the ICU and often in a comatose state, limiting the use of additional assessment metrics. Addressing these limitations is essential for future research improvements in this area.

## 5. Conclusion

EDP therapy demonstrates certain advantages in improving diaphragm function in critically ill patients dependent on mechanical ventilation. EDP can enhance diaphragm strength and endurance, increase diaphragm activity, improve pulmonary ventilation function, and consequently reduce mechanical ventilation duration.

## Author contributions

**Conceptualization:** Linru Qiao, Zhen Yang, Zhixia Zhang.

**Data curation:** Linru Qiao.

**Funding acquisition:** Linru Qiao.

**Investigation:** Linru Qiao, Zhen Yang, Mengli Xu.

**Methodology:** Zhen Yang, Zhixia Zhang.

**Resources:** Zhixia Zhang.

**Software:** Zhen Yang.

**Supervision:** Shangang Zhang.

**Validation:** Shangang Zhang.

**Visualization:** Shangang Zhang.

**Writing – original draft:** Linru Qiao, Zhixia Zhang, Mengli Xu.

**Writing – review & editing:** Linru Qiao, Zhen Yang, Shangang Zhang, Mengli Xu.
